# Estimating causal effects: considering three alternatives to difference-in-differences estimation

**DOI:** 10.1007/s10742-016-0146-8

**Published:** 2016-05-07

**Authors:** Stephen O’Neill, Noémi Kreif, Richard Grieve, Matthew Sutton, Jasjeet S. Sekhon

**Affiliations:** Department of Health Services Research and Policy, London School of Hygiene and Tropical Medicine, London, UK; Manchester Centre for Health Economics, Institute of Population Health, The University of Manchester, Manchester, UK; Department of Political Science and Department of Statistics, University of California at Berkeley, Berkeley, CA USA

**Keywords:** Synthetic control method, Difference-in-differences, Matching, Policy evaluation, Pay-for-performance, I10, I18, C33

## Abstract

Difference-in-differences (DiD) estimators provide unbiased treatment effect estimates when, in the absence of treatment, the average outcomes for the treated and control groups would have followed parallel trends over time. This assumption is implausible in many settings. An alternative assumption is that the potential outcomes are independent of treatment status, conditional on past outcomes. This paper considers three methods that share this assumption: the synthetic control method, a lagged dependent variable (LDV) regression approach, and matching on past outcomes. Our motivating empirical study is an evaluation of a hospital pay-for-performance scheme in England, the best practice tariffs programme. The conclusions of the original DiD analysis are sensitive to the choice of approach. We conduct a Monte Carlo simulation study that investigates these methods’ performance. While DiD produces unbiased estimates when the parallel trends assumption holds, the alternative approaches provide less biased estimates of treatment effects when it is violated. In these cases, the LDV approach produces the most efficient and least biased estimates.

**Electronic supplementary material:**

The online version of this article (doi:10.1007/s10742-016-0146-8) contains supplementary material, which is available to authorized users.

## Introduction

Natural experiments can exploit exogenous variation across time periods and geographical areas to identify the causal effects of alternative policies (Jones and Rice 2011). Difference-in-differences (DiD) methods identify causal effects by contrasting the change in outcomes pre- and post- intervention, for the treatment and control groups (Ashenfelter 1978; Ashenfelter and Card 1985; Bertrand et al. 2004). DiD assumes that, in the absence of treatment, the average outcomes for the treated and control groups would have followed parallel trends over time (Abadie 2005). This assumption allows the averages of the time-invariant unobserved variables to differ between treated and control groups, provided their effects do not change over time. In many health policy settings, the parallel trends assumption is implausible, because unobserved confounders, such as rurality, may have time-varying effects on health outcomes (Ryan et al. 2014). Hence, methods that rely on alternative assumptions warrant consideration.

An alternative set of methods, assume that, in the absence of treatment, the expected outcomes for the treated and control groups would have been the same, conditional on their past outcomes and covariates. This is ‘independence conditional on past outcomes’. This assumption does not require parallel trends, and so allows for the effects of unobserved variables to change over time. This paper considers three approaches that share this assumption: the synthetic control method (Abadie and Gardeazabal 2003; Abadie et al. 2010), a regression method that controls for lagged dependent variables (LDV) (Ashenfelter 1978), and matching directly on past outcomes (Heckman et al. 1997).

The synthetic control method, originally proposed for settings with a single treated unit (Abadie and Gardeazabal 2003; Abadie et al. 2010), has experienced a rapid uptake in the applied program evaluation literature.^1^ This method constructs a comparator, the synthetic control, as a weighted average of the available control units. The weights are chosen to ensure that, prior to the intervention, levels of covariates and outcomes are similar over time to those of the treated unit. While several approaches have been recently proposed to extend the synthetic control method for multiple treated units (Acemoglu et al. 2013; Dube and Zipperer 2013; Kreif et al. 2015; Xu 2015), there are no published simulation studies that examine the relative performance of synthetic control methods versus alternative approaches.

Another approach that avoids the parallel trends assumption is to use multivariate matching (Diamond and Sekhon 2013) to balance the treatment and control groups according to pre-treatment outcomes and covariates (Steventon et al. 2013; Kreif et al. 2015). Applying DiD to the matched data can then control for time-invariant residual biases (Abadie 2005; Blundell and Costa-Dias 2009; Heckman et al. 1997).

A third alternative, is the lagged-dependent-variable approach (LDV), which adjusts for pre-treatment outcomes and covariates with a parametric regression model. The LDV approach has been rarely considered in the program evaluation literature, amid concerns that it can lead to bias if the parallel trends assumption does hold (Angrist and Pischke 2009). The extent to which this concern also applies to the matching and the synthetic control approaches has not been explored. Moreover, in settings where the parallel trends assumption is untenable, there is little empirical evidence to guide the choice between the LDV approach, the synthetic control method and matching on past outcomes (Ryan et al. 2014). A general concern is that it is unknown how these methods perform when faced with relatively few pre-treatment time periods.

An area of high policy relevance where DiD methods have been applied widely is in evaluating pay-for-performance (P4P) schemes for improving health care provision (see for example Eijkenaar 2013; Meacock et al. 2014; Emmert et al. 2012; Lagarde et al. 2013; Epstein 2012; Sutton et al. 2012; Kristensen et al. 2013; Karlsberg-Schaffer et al. 2015). The evidence to support P4P comes predominantly from evaluations that have relied solely on DiD methods. A prime example is the best practice tariffs (BPTs), a hospital P4P scheme introduced in the English NHS from April 2010 for four high-volume clinical conditions. The original evaluation used DiD estimation and reported that BPT had a positive effect on quality and outcome indicators for two of the incentivised conditions (hip fractures and cholecystectomy; McDonald et al. 2012; Allen et al. 2014). However, it is unclear whether the estimated effects were attributable to the P4P scheme or to residual confounding. We reanalyse the BPT scheme for hip fractures, and find that the conclusions from this policy evaluation are sensitive to the choice of method.

We conduct the first Monte Carlo simulation study to contrast the relative performance of DiD compared to these alternative approaches. We consider scenarios where the parallel trends assumption does, and does not hold. The simulation results show that DiD performs best under parallel trends, and when the parallel trends assumption is violated, the LDV approach reports the least biased, most efficient estimates.

The remainder of the paper is organised as follows. In Sect. 2 we introduce the motivating example. Section 3 provides a general overview of the alternative methods, and Sect. 4 contrasts them in the case study. Section 5 presents the methods and results of the simulation study. Section 6 discusses the findings in a broader context, and outlines future research priorities.

## Motivating example: evaluation of a best practice tariffs scheme (BPT)

We re-visit the published evaluation of the BPT scheme for hip fractures (McDonald et al. 2012), which incentivised aspects of clinical practice previously shown to improve health outcomes (Shiga et al. 2008). Participating providers were paid a fixed sum for each hospital admission following hip fracture if certain conditions were met.^2^ The original study contrasted outcomes between 65 participating and 52 non-participating providers. Participation status was defined according to whether the hospital trust had reported receiving any BPT payments for hip fractures in 2010/11 (McDonald et al. 2012). The outcomes of interest were calculated with patient-level data from the Hospital Episode Statistics (HES) database (Health and Social Care Information Centre 2014). These outcomes were: surgery within 48 h; death within 30 days of an emergency admission for hip fracture; emergency re-admission within 30 days of an emergency admission; and return to usual residence within 56 days following admission for hip fracture.

The original DiD analysis reported that the introduction of this BPT led to an increase in the proportion of hip fracture patients receiving surgery within 48 h, of 3.9 percentage points [95 % CI from 2.7 to 5.1 % points], with corresponding changes in 30 day mortality of −0.7 [95 % CI from −1.3 to −0.1], and the proportion of patients discharged to their usual residence of 2.1 [95 % CI from 0.8 to 3.5].

**Fig. 1:**
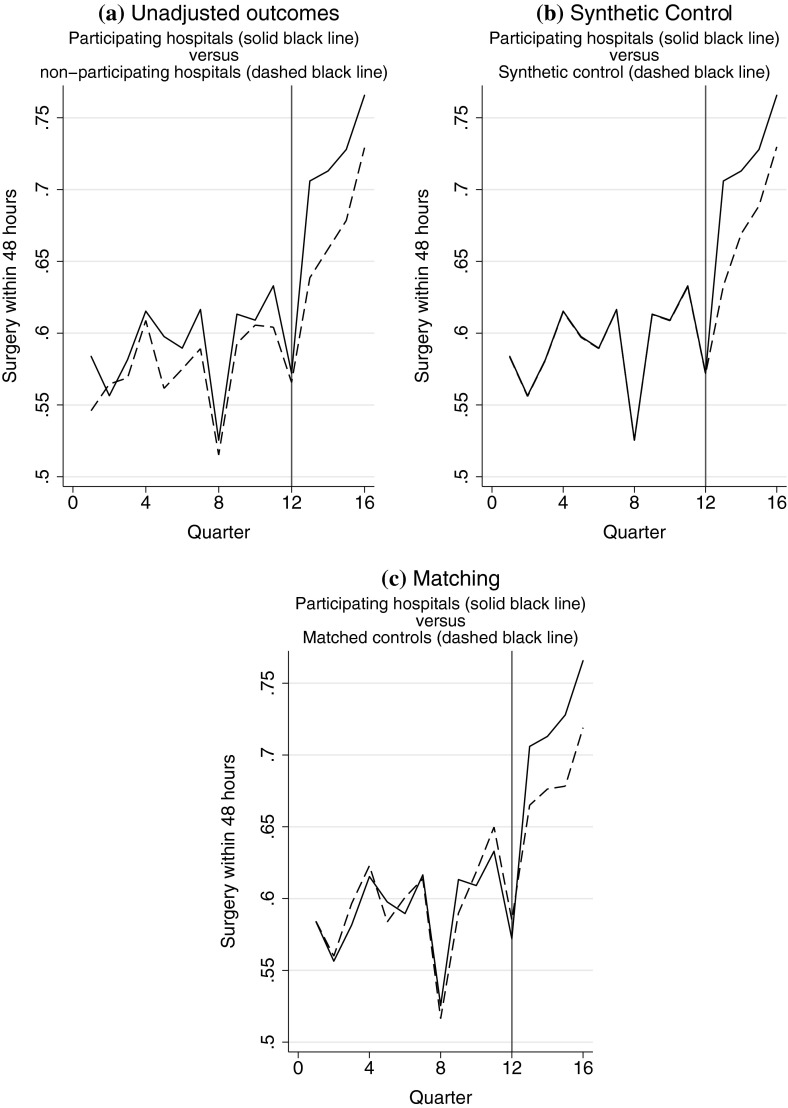
Comparison of surgery within 48 h of emergency admission for hip fracture for participating hospitals to **a** non-participating hospitals, **b** the synthetic control, and **c** the matched controls

**Fig. 2:**
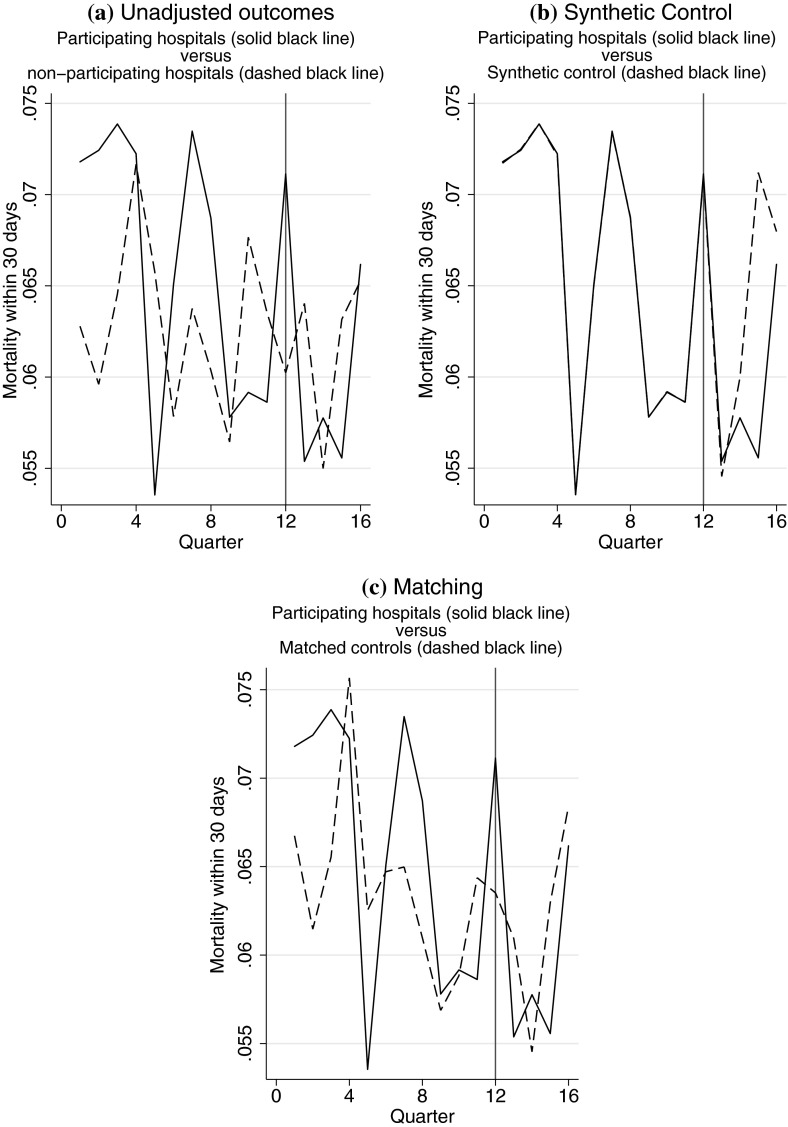
Comparison of mortality within 30 days of emergency admission for hip fracture for participating hospitals to **a** non-participating hospitals, **b** the synthetic control, and **c** the matched controls

The published survey and qualitative interviews undertaken suggested that participation in this BPT scheme was influenced by unobserved factors, such as the resources required for this scheme, which may have had time-varying effects on the outcomes. Hence, a priori, it was unclear whether the parallel trends assumption held. Figure 1a, shows the percentage of patients who had surgery within 48 h, in the 12 quarters before, and four quarters after the scheme’s introduction, which suggests that for this outcome the parallel trends assumption might be reasonable. By contrast, Fig. 2a, shows that for the main outcome, mortality, the parallel trends assumption may be less tenable. Indeed, for death within 30 days the null hypothesis of parallel trends was rejected (*p* = 0.039), although this could not be rejected for the other outcomes. In contrast, the assumption of independence conditional on past outcomes could not be rejected for death within 30 days (*p* = 0.791), while it could be for surgery within 48 h (*p* = 0.001).^3^ However, such tests for parallel trends are not definitive; they only relate to trends in the pre-treatment period, and so alternative methods that avoid this assumption warrant investigation for all endpoints.

## Methods

Throughout we use the potential outcomes framework (Rubin 1974). Suppose there are $i = 1, \ldots ,n$ units (e.g. hospitals), and *T* time periods, where $t = 1, \ldots ,T_{0}$ are pre-treatment, and $T_{0} + 1 , \ldots ..,T$ are post-treatment. The potential outcomes for unit *i* in period *t* in the presence and absence of treatment are denoted by $Y_{it}^{1}$ and $Y_{it}^{0}$ respectively. Let $D_{it}$ be an indicator equal to one if unit *i* is treated in period *t* and zero otherwise. Following Abadie et al. (2010), a general model for the potential outcome in the absence of treatment can be written as:

1 $$Y_{it}^{0} = X_{it} \beta + \lambda_{t} \mu_{i} + \delta_{t} + \varepsilon_{it}$$

where $X_{it}$ is a vector of observed time-varying covariates, $\mu_{i}$ represents time-invariant unobserved characteristics whose effects ($\lambda_{t}$) are assumed not to differ across units but may vary over time, $\delta_{t}$ are common time effects, and $\varepsilon_{it}$ represents exogenous unobserved idiosyncratic shocks. Assuming an additive treatment effect, $\tau_{it}$, we can write the potential outcome under treatment as:

2 $$Y_{it}^{1} = X_{it} \beta + \lambda_{t} \mu_{i} + \delta_{t} + \tau_{it} + \varepsilon_{it}$$

Assuming the treatment only affects the treated units in the periods following treatment, the observed outcome can be written as:

$$Y_{it} = D_{it} Y_{it}^{1} + (1 - D_{it} )Y_{it}^{0}$$

A relevant estimand is the average treatment effect on the treated (ATT) for each post-treatment time period:

$$\tau_{t} = E[Y_{it}^{1} - Y_{it}^{0} |D_{it} = 1]$$

If assignment to the treatment group, and the outcome are both influenced by $\mu_{i}$ (i.e. if $\mu_{i}$ is imbalanced and $\lambda \ne$ 0), then $\mu_{i}$ is an unobserved confounder potentially leading to bias in the estimated ATT.

### Identification of causal effects

To estimate an ATT, it is necessary to make an assumption regarding the outcomes that would have occurred in the absence of treatment ($Y_{it}^{0}$) for the treated units. However, since the true counterfactual outcome cannot be observed in general, the validity of a particular identifying assumption cannot be tested empirically (Imbens and Wooldridge 2009). Here, we consider two distinct identifying assumptions. Firstly, one might assume that the change in $Y_{ }^{0}$ between periods *t* and *t’* is independent of whether the unit is assigned to the treated group, after conditioning on observables (Jones and Rice 2011; Angrist and Pischke 2009). This assumption is commonly referred to as the parallel trends assumption and can be expressed following Abadie (2005) as:

$$E\left( {Y_{it}^{0} - Y_{{it^{\prime}}}^{0} |D_{it} = 1,X_{it} } \right) = E\left( {Y_{it}^{0} - Y_{{it^{\prime}}}^{0} |D_{it} = 0,X_{it} } \right) \quad \left( {{\text{A}}1:{\text{ Parallel trends}}} \right)$$

In the motivating model above (Eq. 1), this requires that the unobserved component, $\lambda_{t} \mu_{i}$, is constant over time (i.e. $\lambda_{t} = \lambda$) if $\mu_{i}$ is imbalanced. Following Jones and Rice (2011) the parallel trends assumption can also be expressed as:

$$Y_{it}^{0} \bot D_{it} |\left( {X_{it} ,t, \lambda \mu_{i} } \right)$$

where the potential outcome under control is assumed to be independent from treatment assignment, conditional on observed confounders, time and individual fixed effects. An alternative, non-nested, assumption is that the treatment-free potential outcome for both groups is the same in expectation conditional on past outcomes (lags) and observed covariates (Angrist and Pischke 2009):

$$Y_{it}^{0} \bot D_{it} |\left( {X_{it} , Y_{ih}^{0} } \right)\quad \left( {{\text{A}}2:{\text{ Independence conditional on past outcomes}}} \right)$$

where ${\text{Y}}_{\text{ih}}^{0}$ is a vector of potential outcomes in the $h$ time periods prior to the introduction of the treatment. Under this assumption, individuals with similar outcomes in the pre-treatment period would be anticipated to have similar potential treatment-free outcomes in post-treatment periods after conditioning on observed covariates $X_{it}$. Thus the two assumptions take alternative views on what is sufficient to condition upon in order to ensure that the treatment-free outcomes are independent of assignment to treatment. In practice, neither of these assumptions may reflect the true treatment-free outcomes of the treated units over time, and so the ATT will not be identified under either assumption. The following sections introduce four estimators, the DiD estimator relying on the first identifying assumption, parallel trends (A1), while the synthetic control, LDV and matching approaches share the second assumption, independence conditional on past outcomes (A2). Assumptions A1 and A2 are non-parametric and do not imply particular model specifications. Where parametric models are used to operationalise these assumptions, there is a risk of model mis-specification which may lead to considerable bias, even if a particular identifying assumption does hold. Throughout this paper we assume that the correct functional form is used when parametrically modelling the impact of observed covariates.

### Estimation

#### Difference-in-differences (DiD)

For the setting with multiple time periods, the following two-way fixed effect regression model can estimate the ATT (Jones and Rice 2011):

3 $$Y_{it} = X_{it} \beta + \lambda \mu_{i} + \delta_{t} + \tau D_{it} + \varepsilon_{it}$$

where $\mu_{i}$ represents unobserved confounders, but, in contrast to the more general model described by Eq. (1), their effects ($\lambda$) are assumed not to vary over time, implying that parallel trends (A1) can be assumed. These unobserved confounders can thus be controlled for by including dummy variables for each unit (individual fixed effects). Common aggregate shocks (δ_t_) can also be controlled for by including dummy variables for each time period (time fixed effects). The estimate for $\tau$ can be interpreted as the ATT averaged across the post-treatment time periods.

If the effects of unobserved confounders on the outcome vary over time (i.e. $\lambda_{t}$ is not constant), this two-way fixed effect model will not in general fully control for bias due to omitted variables.

While more flexible fixed effects specifications are possible, the two-way fixed effects approach is commonly used (Bertrand et al. 2004; Carpenter and Stehr 2008; Fletcher et al. 2015; Wen et al. 2015) and we adopt this model for exposition purposes. We next consider three methods that instead assume independence conditional on past outcomes.

#### Lagged dependent variable approach

The LDV approach estimates the following regression model:

4 $$Y_{it} = X_{it} \beta + \mathop \sum \limits_{k = 1}^{{T_{0} }} \theta_{k} Y_{i,t = k} + \tau D_{i} + \nu_{it} \quad \forall t > T_{0}$$

This model can be estimated using ordinary least squares on the observations in the post-treatment period(s) only. If Eq. (4) represents the true data generating process, then independence conditional on past outcomes (A2) holds and Eq. (4) with $D_{i}$ = 0, represents the counterfactual outcome for the treated unit. Therefore, $\tau$ captures the expected difference between the actual outcome of the treated group and this counterfactual outcome, i.e. the ATT. However, it should be noted that the inclusion of past outcomes here does not create a fully dynamic model since we only condition on a fixed vector of pre-treatment outcomes (${\text{Y}}_{{{\text{i}}h}}$), and not on any lagged outcomes that are post-treatment.^4^

Where instead, Eq. (2) represents the true DGP, the LDV approach may be viewed as proxying the unobserved component ($\uplambda_{\text{t}}\upmu_{\text{i}}$) using a fixed vector of pre-treatment outcomes (${\text{Y}}_{{{\text{i}}h}}$). If the proxies are highly correlated with the unobserved component, bias is expected to be smaller.^5^ The literature on proxy variables suggests that including all available proxies minimises bias (Lubotsky and Wittenberg 2007; Bollinger and Miner 2015). In this case, the inclusion of outcomes for all pre-treatment periods (${\text{Y}}_{{{\text{i}}1}} , \ldots ,{\text{Y}}_{{{\text{i}}T_{0} }}$) is recommended. The LDV approach is expected to perform best when a long pre-treatment period is available. Intuitively, since past outcomes are influenced by unobserved, as well as observed confounders, units with similar past outcomes over an extended period are likely to also be similar in terms of their unobserved confounders (Abadie et al. 2010).

A concern has been raised in the literature that the inclusion of past outcomes as explanatory variables will lead to bias when idiosyncratic shocks are serially correlated (Achen 2000; Keele and Kelly 2006). However, these studies have not focussed on the inclusion of past outcomes as proxies for omitted variables, and they do not consider the estimation of ATT.

#### Synthetic control method

The central idea of the synthetic control method is that the outcomes of the control units can be weighted so as to construct the counterfactual treatment-free outcome for the treated unit. The weights are chosen such that the treated unit and synthetic control have similar outcomes and covariates over the pre-treatment period. Similar to the LDV approach, the synthetic control method also relies on independence conditional on past outcomes (Angrist and Pischke 2009), but takes a semiparametric approach to control these pre-treatment outcomes and covariates, by re-weighting treated observations. In short a synthetic control for a single treated unit is formed by finding the vector of weights W* that minimizes $\left( {X_{1} - X_{0} W} \right)^{\prime } V\left( {X_{1} - X_{0} W} \right)$ subject to the weights in *W* being positive and summing to 1, where X_1_ and X_0_ contain the pre-treatment outcomes and covariates for the treated unit and control units respectively, and ***V*** captures the relative importance of these variables as predictors of the outcome of interest.

For multiple treated units, we follow the approach taken in Kreif et al. (2015), and reweight the disaggregated control units to form an aggregate synthetic control unit. With multiple treated units, X_1_ is the vector of covariates averaged across the treated group. The optimal set of weights creates a synthetic control which approximates the average pre-treatment outcomes ($\bar{Y}_{it} )$ and observed covariates ($\bar{X}_{it} )$ of the treated units:

$$\mathop \sum \limits_{j \in Control} w_{j} Y_{jt} = \bar{Y}_{it} , \quad \forall t \le T_{0}$$

$$\mathop \sum \limits_{j \in Control} w_{j} X_{jt} = \bar{X}_{it} , \quad \forall t \le T_{0}$$

with $0 \le w_{j} \le 1$, and $\mathop \sum \limits_{j \in Control} w_{j} = 1$. If the above holds for a sufficiently long period, it can be assumed that unobserved confounders, and their potentially time-varying effects are also balanced between the synthetic control and the (average) treated unit (Abadie et al. 2010). Under further assumptions, that the data-generating model of the potential outcomes is linear (as in Eq. 1), and the number of pre-treatment periods is large relative to the idiosyncratic shocks ($\varepsilon_{it}$), the difference between the post-treatment outcomes of the treated group and the synthetic control unit has been shown to be an approximately unbiased estimator of the ATT (Abadie et al. 2010).

A potential concern is that when there are few pre-treatment periods relative to the scale of the idiosyncratic shocks, the synthetic control may only appear similar to the treated unit due to these idiosyncratic shocks, leaving imbalances between the comparison groups in time-invariant unobserved confounders. Furthermore, the synthetic control method will generally only assign non-zero weights to a subset of the control pool. This can result in estimates that are inefficient relative to regression approaches, which implicitly use negative weights to construct the counterfactual (Abadie et al. 2010).

#### Multivariate matching combined with DiD

Matching also aims to control for pre-treatment outcomes and covariates, by creating a matched control pool which is similar to the treated group (Heckman et al. 1997; Smith and Todd 2005; Imbens 2004; Stuart et al. 2014). Matching on pre-treatment outcomes may improve balance for the unobserved confounders (μ_i_) with time varying effects to the extent that the outcomes proxy for these confounders. DiD can be subsequently applied to the matched data to try and address any residual imbalances in either time-varying observed confounders or in time-invariant unobserved confounders, and to estimate the ATT.

A matched control group can be created with many alternatives algorithms including nearest neighbour matching, kernel matching, exact coarsened matching or optimal matching, using the propensity score, or multivariate distance measures (Stuart 2010). We use Genetic Matching, a multivariate matching method that explicitly aims to balance the distributions of a pre-specified set of variables, including potential confounders and pre-treatment outcomes (Diamond and Sekhon 2013). Similarly to the synthetic control method, matching is also expected to discard units which are not sufficiently similar to the treated units. Hence, matching followed by DiD may be less efficient than the LDV approach, or DiD used on its own. However, matching is expected to reduce bias from the potential misspecification of the subsequent regression model (Ho et al. 2007).

## Implementing the methods in the re-analysis of BPT for hip fractures

This re-analysis estimates the ATT of participation in the BPT scheme, and considered the same covariates (age group, gender, and source of admission) and outcomes (surgery within 48 h of an emergency admission; death within 30 days; emergency re-admission within 30 days; or return to usual residence within 56 days) as in the original study. The data re-analysed included HES admissions data from 62 hospital trusts that reported receiving at least some BPT payments (treated group), and 49 trusts that reported receiving no payments under the scheme (control group).^6^ Panel data were available for twelve quarters before, and four after, the scheme’s introduction. All subsequent analyses were conducted at the level of the hospital, by quarter.

The DiD estimation was undertaken at the hospital-level and controlled for the above covariates, together with two-way fixed effects for time periods and hospitals. The LDV approach regressed the post-treatment outcomes on the treatment indicator, post-treatment covariates and pre-treatment outcomes as in Eq. (4), using ordinary least squares.

The Synthetic Control method included each covariate averaged over the pre-treatment period and each pre-treatment outcome within X_0_ and X_1_. The multivariate Genetic Matching method matched non-participating to participating hospitals, so as to maximise the balance on pre-treatment outcomes and covariates between the comparison groups, according to paired t-tests and Kolmogorov–Smirnov tests that consider balance according to each variable’s distribution. Just as with the synthetic control method, the algorithm was required to prioritise balance for the pre-treatment outcomes (See Ramsahai et al. 2011). A control unit was matched to each treated unit, with replacement. A two way fixed-effects regression model was then applied to estimate the ATT, with the subsequent inference conditional on the matched data (Ho et al. 2007). All of the regression approaches report standard errors that recognise the clustering of observations within each hospital. ATTs were calculated across the four post-treatment periods, as in the primary analysis. Each method recognised that the number of admissions differed by hospital and quarter, either when weighting the regression model on unmatched data (DiD prior to matching or LDV), creating the aggregate treated unit (synthetic control method), or using patient frequency weights to apply regression to the matched data (matching followed by DiD).

### Case study results

Prior to the introduction of the BPT scheme, the proportion of patients having surgery within 48 h of an emergency admission was generally higher in the participating, than the non-participating hospitals (Fig. 1a); while for mortality, the difference between the two sets of hospitals fluctuates over time (Fig. 2a).^7^

For the prompt surgery endpoint, both the synthetic control and the matching approaches achieved excellent balance (Fig. 1b, c; Table A1). For the mortality outcome, while the synthetic control method achieved good balance (Fig. 2b), matching failed to do so. The standardised differences between the participating and matched non-participating hospitals remained relatively high (greater than 10 %) for several time periods prior to the introduction of the scheme (Table A1; Fig. 2c). The inclusion of pre-treatment outcomes improved the fit of the LDV model (F test for joint significance; *p* < 0.001) supporting the view that the past outcomes are acting as proxies for unobserved potential confounders not already captured by the observed covariates. The ratio of unexplained to explained variation is greater for mortality (4.2) than for surgery (2.2), indicating that the mortality outcome contains a considerable amount of idiosyncratic variation.

Table 1 shows that the alternative approaches to DiD suggest that the BPT led to a greater increase in the proportion of patients having surgery within 48 h, than suggested by the DiD analysis. For mortality within 30 days, DiD reported that the introduction of BPTs led to a 0.8 % points reduction. The alternative approaches all reported a smaller reduction in mortality. Hence the original study’s conclusions are found to be somewhat sensitive to the choice of identifying assumption and estimation approach used.

**Table 1:** BPT case study results: ATT on process and outcome measures according to method

	DiD^a^	LDV	Synthetic controls	Matching + DiD
Surgery within 48 h	0.0403 (*p* = 0.196)	0.0539 (*p* = 0.005)	0.0482 (*p* = 0.250)	0.0488 (*p* = 0.077)
Dead within 30 days	−0.0080 (*p* = 0.037)	−0.0052 (*p* = 0.179)	−0.0051 (*p* = 0.560)	−0.0071 (*p* = 0.052)
Emergency re-admissions, 30 days	0.0003 (*p* = 0.950)	0.0008 (*p* = 0.876)	0.0028 (*p* = 0.775)	0.0047 (*p* = 0.353)
Usual residence, 56 days	0.0228 (*p* = 0.210)	0.0087 (*p* = 0.554)	0.0104 (*p* = 0.655)	0.0124 (*p* = 0.478)

For each method, the analysis adjusted for the following covariates: proportion of patients in age groups defined in 5 year increments from 60 to 105, the proportion of males and the proportion admitted from their usual residence

Reported *p* values are for the null of a true ATT = 0. For DiD and LDV, asymptotic normality is assumed. For Matching +DiD, reported p-values are conditional on the matched data. For Synthetic controls, reported p-values were calculated using placebo-tests in a procedure akin to permutation tests (Abadie et al. 2010). This procedure involves iteratively resampling from the control pool, and in each iteration re-assigning each control unit as a ‘placebo treated unit’, with a probability according to the proportion of treated units in the original sample. The synthetic control method as described in Sect. 3.2.3 was then applied on these ‘placebo data’ and an ATT calculated for the placebo treated versus control units. This iterative process was repeated 200 times, to report a distribution of ATTs under the null hypothesis. The *p* value for the ATT was calculated according to the proportion of the replicates in which the absolute value of the placebo-ATT exceeded the estimated ATT. It should be noted that the *p* value based on placebo tests relate to falsification tests, while the p-values reported for the other methods relate to sampling uncertainty. Hence the *p* values are not directly comparable

^a^McDonald et al. (2012) report similar results for their DiD estimation which was based on patient level data, including year and hospital fixed effects and using robust, unclustered standard errors. Here we conduct the analysis at the hospital trust level using quarterly data, weighting by number of admissions and cluster by hospital trust

## Monte carlo simulation study

### Overview

The simulation study aims to test the following hypotheses raised by the literature review and the case study reanalysis:

1. If the parallel trends assumption holds, DiD estimation will provide the least biased, most precise estimates.
2. When the parallel trends assumption fails, the LDV, synthetic control method and matching combined with DiD will lead to less bias than DiD alone, *if* the past outcomes proxy the time-varying effects of the unobserved confounders.
3. The synthetic control method, and matching combined with DiD, are expected to be relatively inefficient compared to the LDV approach.
4. When idiosyncratic shocks are serially correlated, the inclusion of past outcomes as explanatory variables will increase bias (Achen 2000; Keele and Kelly 2006).
5. With few time periods, and high variance of the idiosyncratic shocks, the methods relying on independence conditional on past outcomes for identification are anticipated to lead to greater bias (Abadie et al. 2010).

### Data generating process

We conduct Monte Carlo simulation studies where the true ATT is known and contrast the four approaches in terms of their bias (%) and Root Mean Squared Error (RMSE).

We created 1000 datasets, each with 150 units, of which 75 were assigned to treatment in the last time period. As in Abadie et al. (2010), the data generating process (DGP) includes an unobserved component with an effect that changes over time ($\lambda_{t} \mu_{i}$):

$$Y_{it} = X_{1,it} \beta_{1} + X_{2,it} \beta_{2} + \lambda_{t} \mu_{i} + D_{it} \tau + \varepsilon_{it}$$

The observed covariates $X_{1,it}$, $X_{2,it}$ and an unobserved confounder, $\mu_{i}$, are generated from correlated normal distributions. To introduce imbalance between the treated and control groups, the means of $X_{1,it}$, $X_{2,it}$ and $\mu_{i}$ are set one standard deviation higher for the treated units than for the controls. $\varepsilon_{it}$ is a normally distributed idiosyncratic error term with mean zero and standard deviation $\sigma_{\varepsilon }$. The parallel trends assumption holds when $\lambda_{t}$ is constant, and fails when it is allowed to vary over time. As health data often exhibits a trend and seasonal component, in scenarios where the parallel trends fails, we allow $\lambda_{t}$ to consist of a constant, a time trend and a seasonal cycle which is represented by a sinewave.

The DGP above does not include pre-treatment outcomes on the right hand side, that is, the simulation does not include a scenario where independence conditional on past outcomes holds exactly. Rather the methods that rely on this assumption use lagged outcomes to proxy the effects of unobserved confounders ($\lambda_{t} \mu_{i}$) (see Appendix A for further details).

### Simulation scenarios

We consider four main scenarios (see Table 2**)**. In Scenario A the parallel trends assumption holds, ($\lambda$ is constant), whereas in Scenarios B–D the PT assumption fails $(\lambda_{t}$ varies over time). Scenarios A, B and D assume no serial correlation for the idiosyncratic shock, whereas Scenario C assumes a high positive level of serial correlation (ρ = 0.7)^8^ (see Appendix B for Scenarios C1, C2 and C3 with levels of serial correlation of −0.7, 0.4 and −0.4 respectively). Scenario D considers an outcome with high variance—a case that is anticipated to prove challenging for all methods that rely upon past outcomes being a proxy for the effect of time-varying confounders.^9^ For each scenario we conduct simulations using 3, 10 and 30 periods, with the final period considered to be post-treatment.

**Table 2:** Monte Carlo simulations: summary of parameter values across the scenarios

Scenario	Scenario description	Total periods	Std. deviation of epsilon ($\sigma_{\varepsilon }$)	Settings for λ			Serial correlation ($\uprho$)
				Trend $\left( {\delta_{t} } \right)$	Amplitude (*A*)	Wave length (*w*)	
A	Parallel trends holds	{3, 10, 30}	10	0	0	0	0
B	Parallel trends fails	{3, 10, 30}	10	10	2	4	0
C	Parallel trends fails + serial correlation	{3, 10, 30}	10	10	2	4	0.7
D	Parallel trends fails + high variance	{3, 10, 30}	50	10	2	4	0

Across all scenarios: effect of covariates ($\beta_{j} ) = 1$ and Average Treatment effect $(\tau ) = 10.$ Serial correlation: $\varepsilon_{it} =\uprho \times \varepsilon_{it - 1} + N(0,\sigma_{\varepsilon }$)

Time-varying effect of unobserved confounders: $\lambda_{t} = \left( {1 + \delta_{t} \left( {1 - \frac{{\left( {t - T} \right)}}{50}} \right) + A \times \sin \left( {\frac{2\pi }{w}} \right)} \right)$

Finally, we also consider alternative specifications for λ_t_ where (a) the trend in λ_t_ is quadratic rather than linear (Scenario E) or (b) where λ_t_ is a constant in the pre-treatment period and a different constant in the post-treatment period (Scenario F).

### Simulation results

**Fig. 3:**
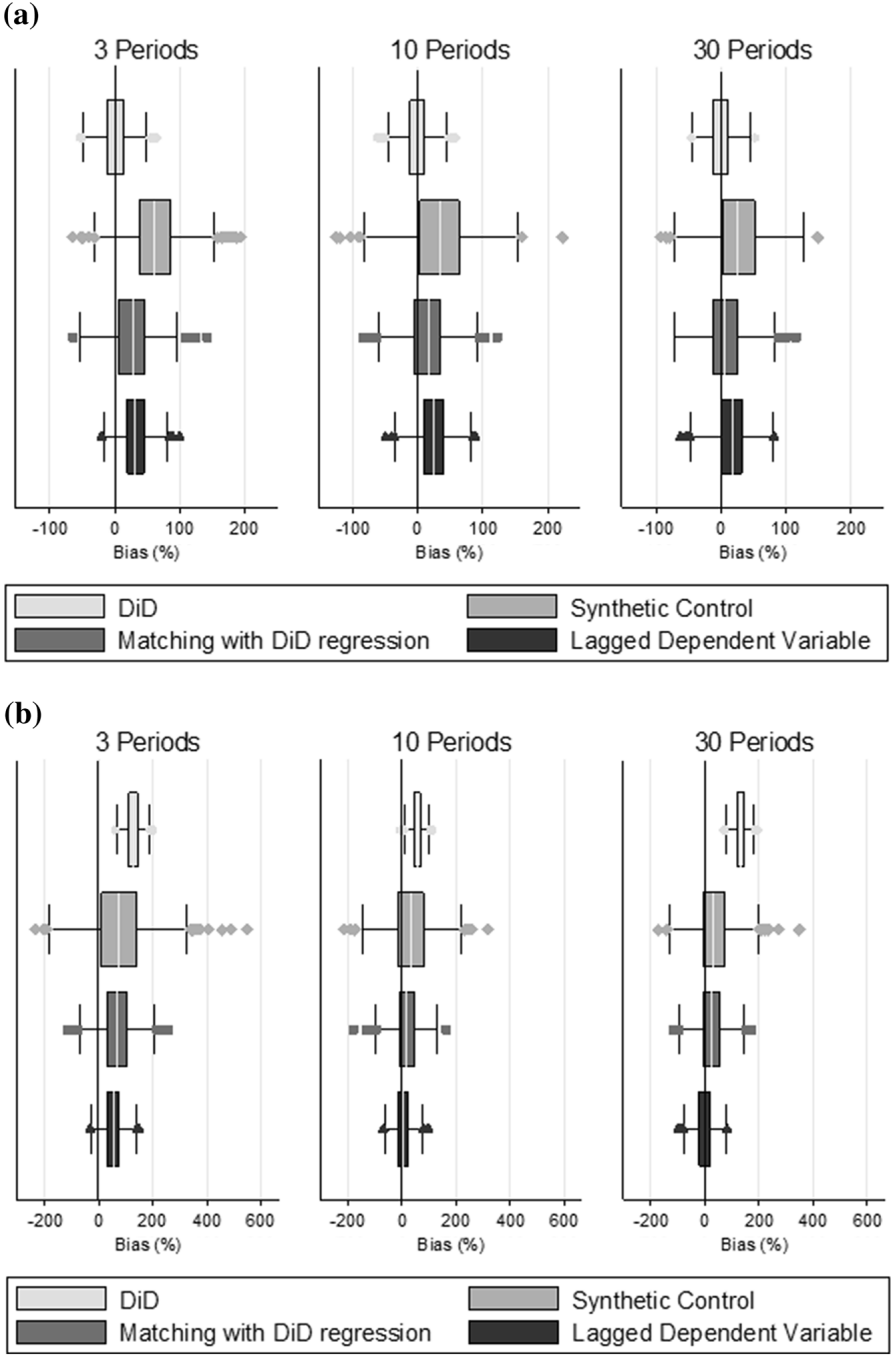

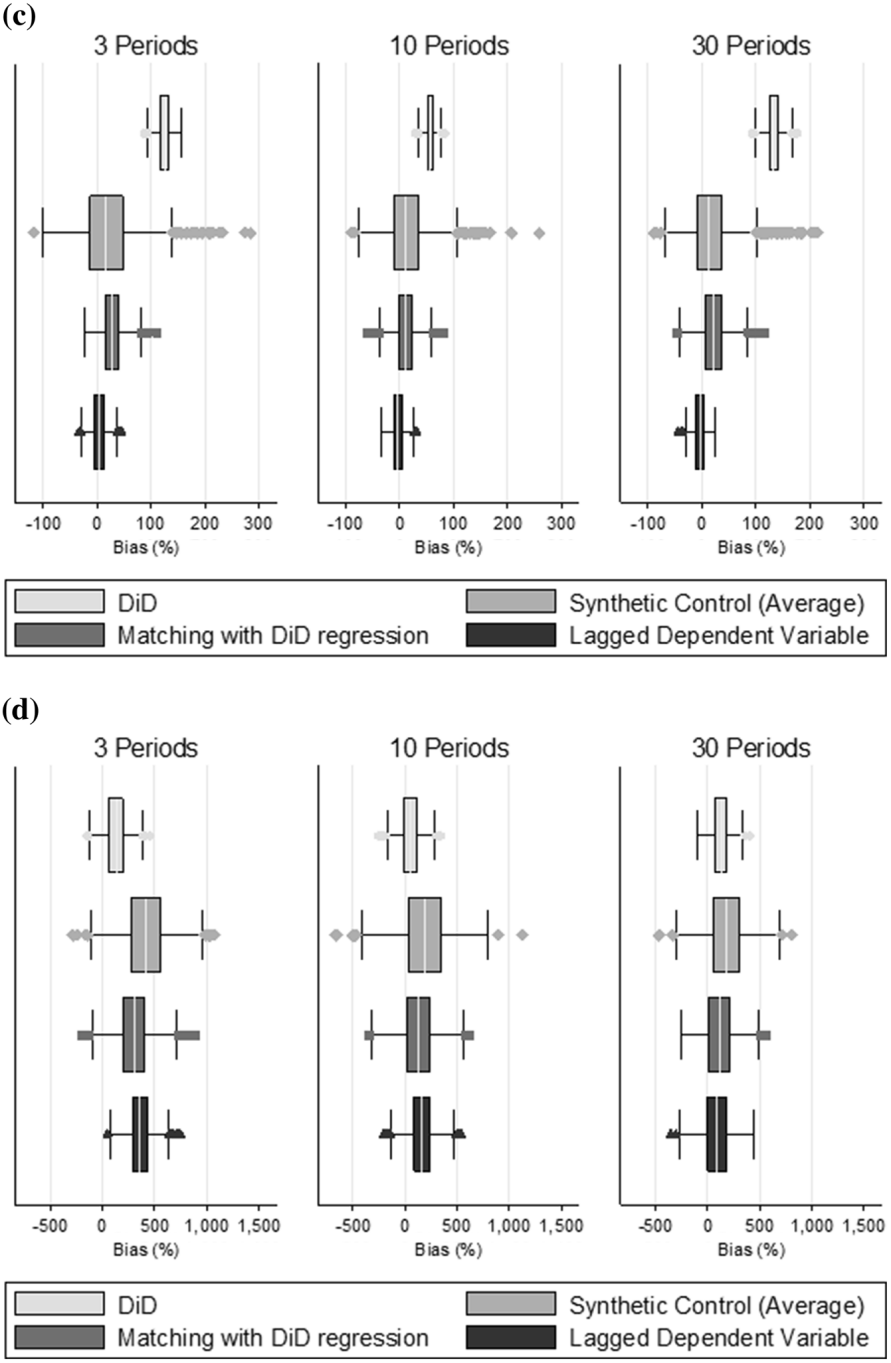
Monte Carlo simulation results: bias (%) and distribution of the estimates: **a** Scenario A—parallel trends. **b** Scenario B—non parallel trends, no serial correlation (ρ = 0), low outcome variation (σ_e_ = 10). **c** Scenario C—non parallel trends and high serial correlation (ρ = 0.7). **d** Scenario D—non parallel trends and high outcome variation (σ_e_ = 50)

Figure 3 summarises the estimates from the Monte Carlo simulation and Table 3 presents percentage bias and RMSE. Where the parallel trends assumption holds (scenario A) DiD estimates have the lowest bias and RMSE (Fig. 3a). The other methods report biases of between 10 and 30 %, with larger bias in scenarios with few (two) pre-treatment time periods. The synthetic control method reported higher RMSE than the other approaches.

**Table 3:** Monte Carlo simulation: bias (%) and RMSE for estimation of the ATT (true value of 10)

Scenario	Description	Periods	Bias (%)			RMSE		
			3	10	30	3	10	30
A	Parallel trends holds	DiD	1	−1	−1	2	2	2
		Synthetic controls	63	33	26	7	6	5
		LDV	32	23	16	4	3	3
		Matching + DiD	27	16	7	4	3	3
B	Parallel trends fails	DiD	127	57	132	13	6	13
		Synthetic controls	75	34	37	13	8	8
		LDV	53	5	−2	6	3	3
		Matching + DiD	69	18	26	9	5	5
C	Parallel trends fails + serial correlation (ρ = 0.7)	DiD	127	57	132	13	6	13
		Synthetic controls	23	17	20	6	4	5
		LDV	5	−3	−4	1	1	1
		Matching + DiD	29	12	21	4	2	3
D	Parallel trends fails + high variance	DiD	129	52	128	16	10	15
		Synthetic controls	419	189	176	47	30	25
		LDV	355	165	90	37	20	16
		Matching + DiD	301	124	106	34	20	18

The reason that matching combined with DiD reports increased bias, despite parallel trends holding, can be explained as follows. While matching on past outcomes and covariates in a particular period *k*, ensures that the treated and matched controls have (on average) similar values for the combined unobserved term $\left( {\lambda \mu_{i} + \varepsilon_{ik} } \right)$, it does not ensure that the units are well matched in terms of $\mu_{i}$, rather the units may only appear to be similar due to the ‘noise’, $\varepsilon_{ik}$. As E($\mu_{i}$) is greater for the treated units than for the controls, matching tends to select those control units that have positive values for $\varepsilon_{ik}$. In the post-treatment period, *t*, the shocks ($\varepsilon_{it}$) of the matched controls tend to their mean of 0, and so even in the absence of treatment, the matched units will not be similar, introducing bias. As the number of periods over which the units are matched increases, a series of positive idiosyncratic shocks becomes increasingly less likely, and so matching is more likely to ensure similar $\mu_{i}$, and hence the bias is reduced.

When the parallel trends assumption fails (Scenario B), DiD reports estimates with low variation, but high bias (Fig. 3b). By contrast, the synthetic control method, and matching combined with DiD estimation provide ATT estimates with low bias but higher variance, while the LDV approach reports the lowest bias and RMSE (Fig. 3b; Table 3). This supports hypotheses 2 and 3.

In Scenario C (Fig. 3c), where idiosyncratic shocks are strongly positively correlated, the three methods that assume independence conditional on past outcomes report less bias than in Scenario B (uncorrelated shocks), while as expected the bias for DiD is unaffected. One explanation is that λ_t_μ_i_ is also positively serially correlated here, since λ_t_ includes a time trend. Therefore units which appear similar in the pre-treatment periods will be more similar when there is positive serial correlation in the idiosyncratic shocks, than when there is no serial correlation. While the sign and level of serial correlation influences the performance of the LDV, synthetic control and matching on past outcomes approaches, the LDV approach continues to offer the best performance of these alternatives and its performance improves as the number of pre-treatment periods increases (Scenarios C1–C3, Table A3).

In Scenario D (Fig. 3d), with a high variance of the idiosyncratic shocks, all methods perform poorly, with bias of between 50 and 420 %. For the LDV, synthetic control and matching combined with DiD, this is attributable to past outcomes being less informative about time invariant unobservables, and bias reduced as the number of pre-treatment periods increased. In contrast, for DiD the bias is due to the violation of the parallel trends assumption and is similar in magnitude to the bias observed in Scenario B.

Under alternative specifications of $\lambda_{t} ,$ the LDV continues to perform relatively well in terms of both efficiency and bias (see Table A4, Figures A4a and A4b and A5 in Appendix B).

## Discussion

This paper presents the first simulation study to assess the relative performance of DiD compared to the synthetic control, matching and LDV approaches. Where the parallel trends assumption is violated, we find that DiD provides biased estimates while the synthetic control approach mitigates this bias. In line with the theoretical results outlined by Abadie et al. (2010), increasing the number of pre-treatment periods further reduces the bias reported by the synthetic control approach. However, the estimates using synthetic controls are relatively inefficient. The LDV approach returns more efficient estimates than the synthetic control approach, while also further mitigating bias. We conclude that the LDV approach is an attractive estimation approach in this setting, provided the functional form for observed covariates is correctly specified.

Angrist and Pischke (2009) stress that the assumptions underlying the DiD and the LDV approaches are not nested, and that including lagged dependent variables can induce bias when the parallel trends assumption is actually correct. Our findings are in line with this, and we also show that the synthetic control and matching approaches report greater bias than LDV, when the parallel trends assumption holds.

The good performance of the LDV approach can be explained by the ability of the lagged outcomes to proxy for the effects of the omitted unobserved confounder. It should be recognised that we designed the simulations so that when the parallel trends assumption fails, due to the presence of time-varying effects of the unobserved confounder, none of the methods are correctly specified. We find that this result holds across a variety of ways in which the unobserved confounders enter the true DGP. In contrast to the prevailing view in the literature on models that include past outcomes (Achen 2000; Keele and Kelly 2006; Kayser and Wlezien 2011; Balaev 2014), we find that serial correlation does not increase the bias of the LDV approach and in fact may improve performance in some cases. The simulation study also suggests that the effects of serial correlation diminish as the number of pre-treatment periods increases. In the case study, serial correlation is of little concern since mortality does not appear to be serially correlated, while for surgery, the parallel trends assumption is tenable, allowing the use of DiD, whose point estimates are not affected by serial correlation.

We find that no method reports unbiased estimates in all settings. Since the identifying assumptions of the methods are inherently untestable, the failure of any method to report unbiased estimates across all simulation scenarios argues in favour of presenting results based on alternative methods. As our re-analysis of the BPT case study shows, such sensitivity analyses can be important in communicating to policy-makers that policy conclusions can be sensitivity to the choice of method. The insights from both the re-analysis of the BPT example and the simulation study, highlights the need for careful consideration of the underlying assumptions of the methods used. Our results suggest that future studies should extend the time period over which pre-intervention outcome data are collected, to reduce bias when using methods that rely on these data for identification.

We caution policy-makers against drawing firm conclusions from analysis that solely relies on either one of these identification assumption (parallel trends or conditional on lagged outcomes), in settings where there is not definitive evidence that either of the identification assumptions is supported for all the endpoints of interest (as per the BPT example). In many settings, the available evidence may not provide strong support for either of these assumptions, and so our general recommendation is that the base case analysis should present results from the method(s) that uses the ‘most plausible’ identification assumption, but then the sensitivity analysis should present findings from method(s) that make alternative, but still ‘somewhat plausible’, identification assumptions. In the absence of a strong justification for either identification assumption, we recommend reporting results under alternative assumptions and acknowledging that they do not offer a strong basis for causal inference.

This paper has the following limitations. First, each of the methods considered assumes that any idiosyncratic shocks following the introduction of the intervention have the same expected effect on outcomes for the treated and control groups. Second, in the interests of simplicity and transparency, the data generating process in the simulation study assumed that the observed and unobserved covariates all have a linear additive effect on the outcome. It is important to note that even when their respective identification assumptions hold, if the functional form assumptions underlying the estimators are violated then each of these methods can lead to biased estimates.

The relative performance of matching may improve with a less restrictive DGP. Finally, it was not feasible to consider the full range of modelling approaches available. While the inclusion of unit specific trends in a DiD model (Bell et al. 1999; Wagstaff and Moreno-Serra 2009) may perform well when the trends are readily apparent from the data, correctly specifying unit specific trends may prove challenging. Particularly in small samples, where the outcome is noisy, or data are only available for a limited number of pre-treatment periods, this may lead to over-fitting and hence introduce bias.

A further limitation of this work is that we restrict our attention to two alternative identifying assumptions. Other approaches, relying on alternative identifying assumptions may also warrant consideration in contexts beyond those considered in this paper. For instance marginal structural models (MSMs), which typically assume independence conditional on included covariates, and rely on the correct specification of the treatment assignment mechanism (the propensity score) for inverse probability weighting (Cole and Hernán 2008), have proven useful in contexts where treatment receipt changes over time.

This paper provokes several areas for further research. First, the DGP could be extended to consider a broader range of scenarios including imbalances in higher moments of the covariate distributions, and non-linear effects of observed and unobserved confounders on the outcome. Such scenarios are likely to reveal improved performance by the multivariate matching approach which can reduce imbalance in moments of the distribution beyond the mean and would be less sensitive to functional form misspecification (Ho et al. 2007). Second, the LDV model could be estimated with more flexible regression methods, such as the lasso to penalise over-fitting (Tibshirani 1996), potentially combined with nonlinear terms. Third, the recently proposed generalised synthetic control method (Xu 2015), which uses linear interactive fixed effect models to impute the potential outcomes under control, warrants further consideration.

## Electronic supplementary material

Below is the link to the electronic supplementary material.

Supplementary material 1 (DOCX 55 kb)

Supplementary material 2 (EPS 3133 kb)

Supplementary material 3 (EPS 3129 kb)

Supplementary material 4 (EPS 68 kb)

Supplementary material 5 (EPS 2893 kb)

Supplementary material 6 (EPS 2893 kb)

Supplementary material 7 (EPS 2892 kb)

## Footnotes

1 The paper outlining the synthetic control method by Abadie et al. (2010) has been cited 718 times (Google Scholar, accessed 23rd December, 2015).

2 These conditions are: first, the patient had surgery within 36 h following presentation at the emergency department or clinical diagnosis (inpatient admissions); second, an ortho-geriatrician attended the pre-operative assessment, and directed the patient’s post-operative rehabilitation and discharge planning. To be eligible for the BPT bonus, the provider had to agree with their local purchaser that the bonuses would be paid, in a manner similar to the design of the local Commissioning for Quality and Innovation (CQUIN) payments (Kristensen et al. 2013).

3 Further details on these tests are provided in Appendix C.

4 In a dynamic model, the vector of past outcomes, is defined relative to the period being considered. In contrast, here the vector of past outcomes is the same regardless of the period considered.

5 In fact the inclusion of fixed effects in DiD can also be viewed as proxying for unobserved confounders since the fixed effects generally do not directly impact on outcomes but rather control for time-invariant unobserved variables. The DiD approach thus assumes that including individual and period specific fixed effects is sufficient to proxy for all confounders. The LDV approach instead assumes that the pre-treatment outcomes are sufficient proxies.

6 A small number of observations (32 out of 1808) were excluded as data for these hospital trusts was not available for the full period considered. A further 16 observations were excluded when considering return to usual residence due to missing data for this measure.

7 For readmissions and return to usual residence, a similar pattern as with surgery was found.

8 Note that in the case study, the null hypothesis of no serial correlation cannot be rejected for mortality (*p* = 0.4666), readmissions (*p* = 0.4998) and return to usual residence (*p* = 0.8414), while for time to surgery the null is rejected (*p* = 0.0045), however the serial correlation for the estimated residuals for this outcome is still relatively weak (0.25).

9 The ratio of unexplained to explained variation in the BPT evaluation is closer to that in Scenario A and B. The ratio of unexplained to explained variation in Scenarios A to D were approximately 2, 2, 1 and 11 respectively.
