# Supplementary material for: Estimating causal effects: considering three alternatives to difference-in-differences estimation
Source: Health Serv Outcomes Res Methodol. 2016 May 7;16:1–21. doi: 10.1007/s10742-016-0146-8 (PMC4869762; doi:10.1007/s10742-016-0146-8)
Supplement: Supplementary file 1 — Supplementary material 1 (DOCX 55 kb) [file 10742_2016_146_MOESM1_ESM.docx]

# Online Appendix A - Overview of Simulation Study

To assess the relative performance of the different approaches discussed previously, we conduct a set of Monte Carlo simulation studies where the true ATT is known and contrast the approaches in terms of their bias and Mean Squared Error (MSE).

$$Y_{it}=X_{it}\beta+\tau\left( D\times Post \right)+\lambda_{t}\mu_{i}+\varepsilon_{it}$$

1. First, 150 units are created with 75 assigned to the treatment group ($D=1$) and 75 not ($D=0$).
2. Time-invariant variables $\left( X_{1i},X_{2i},\mu_{i}, \right)$ are drawn from a standard multivariate normal distribution with the correlation between $X_{1i} \mathrm{and}X_{2i}$ = 0 and between $X_{1i} \mathrm{or} X_{2i}\mathrm{and}\mu_{i}$ = $\rho_{X\mu}$.
3. $\mu_{i}$ is then rescaled to have a mean of 5 and a standard deviation ($\sigma_{\mu}$) of 2.
4. These time-invariant variables are then replicated for *T* periods and a dummy variable, *Post*, is created to represent the post-treatment period, which consists of only the final period in the simulations considered here.
5. Next for each period (i.e. *t*) time-varying components of the observed covariates ($X_{1it} \mathrm{and}X_{2it}$) are drawn from standard normal distributions.
6. The time-invariant and time-varying parts of the X variables are then combined and rescaled: $X_{k}=\left( X_{ki}+X_{kit} \right)*s_{X}+E(X)$ where $E(X)=4\mathrm{and}s_{X}=2$ for k={1,2}.
7. In scenarios with imbalance in the observed covariates, the distribution of each variable ($X_{k}$) for the treated group was shifted upwards by $s_{X}$. Similarly $\mu_{i}$ was shifted upwards by $\sigma_{\mu}$ for the treated group to reflect imbalance in the unobserved covariates where applicable.
8. $\lambda_{t}$, the effect of the unobserved covariates ($\mu_{i}$), are generated as a combination of a constant, a linear trend and a sinewave: $\lambda_{t}=\left( 1+\delta\left( 1-\frac{\left( t-T \right)}{50} \right)+A*\sin\left( \frac{2\pi}{w} \right) \right)$. Where *A* is the amplitude ($A=2$), *w* is the wavelength ($w=4$) and $\delta$ determines the slope of the linear trend $\left( \delta=10 \right)$ while the inclusion of $\left( 1-\frac{\left( t-T \right)}{50} \right)$ ensures that the maximum value of $\lambda_{t}$ does not change as the number of periods considered (*T*) changes.
9. Finally a normally distributed idiosyncratic shock, $\varepsilon_{it}$, with mean 0 and standard deviation $\sigma_{\varepsilon}$ is created.
10. The variables created in steps (1) to (9) are then combined to generate the observed outcome variable: $Y_{it}=X_{it}\beta+\tau\left( D\times Post \right)+\lambda_{t}\mu_{i}+\varepsilon_{it}$.

The process in steps (1) to (10) is repeated to create 1,000 datasets for each simulation scenario using the relevant parameters for that scenario.

# Online Appendix B – Additional Tables & Figures

**Table A1: Comparison of balance for outcomes prior to the introduction of BPT scheme before- and after multivariate matching (Genetic Matching)**

1. surgery within 48 hours of emergency admission for hip fracture

|  |  | Before matching |  |  |  | After matching |  |
| --- | --- | --- | --- | --- | --- | --- | --- |
| Number of quarters pre intervention | Participating hospitals | Non-participating hospitals | Standardised difference |  | Participating hospitals | Non-participating hospitals | Standardised difference |
|  | % | % | % |  | % | % | % |
| 12 | 60.6 | 56.5 | 22.2 |  | 60.6 | 60.2 | 2.6 |
| 11 | 58.9 | 59.0 | -0.5 |  | 58.9 | 59.0 | -0.5 |
| 10 | 61.8 | 58.9 | 16.1 |  | 61.8 | 62.3 | -3.3 |
| 9 | 63.7 | 63.4 | 2.0 |  | 63.7 | 64.1 | -3.3 |
| 8 | 62.0 | 60.2 | 12.1 |  | 62.0 | 62.1 | -0.4 |
| 7 | 62.8 | 60.8 | 12.2 |  | 62.8 | 63.2 | -2.9 |
| 6 | 63.9 | 61.9 | 12.5 |  | 63.9 | 63.2 | 4.2 |
| 5 | 54.2 | 53.1 | 7.2 |  | 54.2 | 54.1 | 0.7 |
| 4 | 62.4 | 61.4 | 6.5 |  | 62.4 | 62.2 | 1.3 |
| 3 | 63.6 | 62.2 | 9.0 |  | 63.6 | 64.1 | -3.4 |
| 2 | 66.8 | 62.9 | 27.9 |  | 66.8 | 66.1 | 5.3 |
| 1 | 58.6 | 56.8 | 12.4 |  | 58.6 | 59.7 | -7.3 |

1. mortality within 30 days of emergency admission for hip fracture

|  |  | Before matching |  |  |  | After Matching |  |
| --- | --- | --- | --- | --- | --- | --- | --- |
| Number of quarters pre-intervention | Participating hospitals | Non-participating hospitals | Standardised difference |  | Participating hospitals | Non-participating hospitals | Standardised difference |
|  | % | % | % |  | % | % | % |
| 12 | 7.5 | 6.5 | 25.4 |  | 7.5 | 6.8 | 16.5 |
| 11 | 7.9 | 6.1 | 27.5 |  | 7.9 | 6.2 | 26.3 |
| 10 | 7.2 | 6.3 | 19.6 |  | 7.2 | 6.5 | 14.8 |
| 9 | 7.5 | 7.3 | 3.6 |  | 7.5 | 7.3 | 3.6 |
| 8 | 5.6 | 6.5 | -28.4 |  | 5.6 | 6.5 | -28.9 |
| 7 | 6.1 | 5.6 | 13.1 |  | 6.1 | 6.5 | -8.3 |
| 6 | 7.0 | 6.5 | 10.7 |  | 7.0 | 6.8 | 4.1 |
| 5 | 7.3 | 6.1 | 26.3 |  | 7.3 | 6.2 | 24.1 |
| 4 | 5.1 | 6.0 | -24.4 |  | 5.1 | 6.1 | -27.4 |
| 3 | 6.1 | 6.9 | -18.6 |  | 6.1 | 5.8 | 5.4 |
| 2 | 5.8 | 6.0 | -6.0 |  | 5.8 | 6.4 | -16.2 |
| 1 | 6.8 | 6.2 | 15.0 |  | 6.8 | 6.5 | 7.3 |

**Table A2: Estimated coefficients from regression models for DiD pre and post matching and for LDV model for:**

1. Surgery within 48 hours of emergency admission for hip fracture

| **Variable** | **DiD** | | **Matching+DiD** | | **LDV** | |
| --- | --- | --- | --- | --- | --- | --- |
|  | **Coefficient** | **Std. error** | **Coefficient** | **Std. error** | **Coefficient** | **Std. error** |
| **ATT** | 0.0403 | (0.015) | 0.0488* | (0.027) | 0.0539*** | (0.011) |
| **Proportion of patients:** |  |  |  |  |  |  |
| Age group 65 | -0.255 | (0.287) | -0.159 | (0.266) | 0.209 | (0626) |
| Age group 70 | -0.168 | (0.285) | -0.164 | (0.266) | 0.390 | (0.608) |
| Age group 75 | -0.240 | (0.276) | -0.076 | (0.258) | 0.413 | (0.607) |
| Age group 80 | -0.113 | (0.273) | -0.122 | (0.248) | 0.422 | (0.593) |
| Age group 85 | -0.126 | (0.272) | -0.125 | (0.250) | 0.550 | (0.596) |
| Age group 90 | -0.146 | (0.275) | -0.112 | (0.251) | 0.582 | (0.595) |
| Age group 95 | -0.219 | (0.278) | -0.197 | (0.250) | 0.570 | (0.596) |
| Age group 100 | -0.091 | (0.289) | 0.006 | (0.291) | 0.626 | (0.625) |
| Age group 105 | -0.555 | (0.436) | -0.579* | (0.395) | 1.218 | (0.925) |
| Admitted from usual residence | -0.093 | (0.122) | 0.014 | (0.124) | -0.048 | (0.108) |
| Male | -0.015 | (0.048) | -0.019 | (0.047) | 0.012 | (0.101) |
| Qu­­arter 2 | -0.003 | (0.017) | -0.023* | (0.012) |  |  |
| Quarter 3 | 0.010 | (0.015) | 0.007 | (0.010) |  |  |
| Quarter 4 | 0.048*** | (0.015) | 0.039*** | (0.011) |  |  |
| Quarter 5 | 0.014 | (0.015) | 0.005 | (0.014) |  |  |
| Quarter 6 | 0.019 | (0.015) | 0.017 | (0.013) |  |  |
| Quarter 7 | 0.041*** | (0.015) | 0.032** | (0.014) |  |  |
| Quarter 8 | -0.041*** | (0.014) | -0.057*** | (0.013) |  |  |
| Quarter 9 | 0.039*** | (0.015) | 0.019 | (0.014) |  |  |
| Quarter 10 | 0.042** | (0.015) | 0.036** | (0.014) |  |  |
| Quarter 11 | 0.057*** | (0.015) | 0.063*** | (0.014) |  |  |
| Quarter 12 | 0.009 | (0.016) | 0.003 | (0.0.16) |  |  |
| Quarter 13 | 0.090*** | (0.017) | 0.082*** | (0.016) |  |  |
| Quarter 14 | 0.104*** | (0.018) | 0.092*** | (0.017) |  |  |
| Quarter 15 | 0.122*** | (0.018) | 0.100*** | (0.017) |  |  |
| Quarter 16 | 0.163*** | (0.020) | 0.137*** | (0.017) |  |  |
| **Outcome *k* periods prior to BPT introduction:** | |  |  |  |  |  |
| k=12 |  |  |  |  | -0.002 | (0.062) |
| k=11 |  |  |  |  | -0.055 | (0.063) |
| k=10 |  |  |  |  | -0.039 | (0.079) |
| k=9 |  |  |  |  | 0.063 | (0.063) |
| k=8 |  |  |  |  | 0.034 | (0.071) |
| k=7 |  |  |  |  | -0.081 | (0.067) |
| k=6 |  |  |  |  | 0.065 | (0.066) |
| k=5 |  |  |  |  | -0.060 | (0.066) |
| k=4 |  |  |  |  | 0.117 | (0.063) |
| k=3 |  |  |  |  | 0.119 | (0.071) |
| k=2 |  |  |  |  | -0.053 | (0.077) |
| k=1 |  |  |  |  | 0.352*** | (0.062) |
| Intercept | 0.815 | (0.291) | 0.696*** | (0.261) | -0.065 | (0.599) |
| No. of observations | 1776 |  | 1984 |  | 444 |  |

1. mortality within 30 days of emergency admission for hip fracture

| **Variable** | **DiD** | | **Matching+DiD** | | **LDV** | |
| --- | --- | --- | --- | --- | --- | --- |
|  | **Coefficient** | **Std. error** | **Coefficient** | **Std. error** | **Coefficient** | **Std. error** |
| **ATT** | -0.0080** | (0.003) | -0.0071* | (0.004) | -0.0052 | (0.003) |
| **Proportion of patients** |  |  |  |  |  |  |
| Age group 65 | -0.089 | (0.089) | -0.139 | (0.089) | -0.255 | (0.173) |
| Age group 70 | -0.173* | (0.087) | -0.197** | (0.090) | -0.316* | (0.168) |
| Age group 75 | -0.137 | (0.086) | -0.192** | (0.088) | -0.228 | (0.168) |
| Age group 80 | -0.122 | (0.086) | -0.196** | (0.085) | -0.291* | (0.164) |
| Age group 85 | -0.074 | (0.085) | 0.118 | (0.088) | -0.214 | (0.164) |
| Age group 90 | -0.064 | (0.086) | 0.111 | (0.085) | -0.244 | (0.163) |
| Age group 95 | -0.010 | (0.086) | -0.054 | (0.086) | -0.153 | (0.164) |
| Age group 100 | 0.046 | (0.092) | -0.015 | (0.092) | -0.162 | (0.172) |
| Age group 105 | 0.072 | (0.128) | 0.009 | (0.129) | 0.066 | (0.257) |
| Admitted from usual residence | 0.050* | (0.024) | 0.044 | (0.035) | 0.025 | (0.029) |
| Male | 0.051*** | (0.014) | 0.040*** | (0.014) | 0.088*** | (0.028) |
| Quarter 2 | -0.001 | (0.004) | -0.003 | (0.004) |  |  |
| Quarter 3 | 0.001 | (0.004) | -0.001 | (0.004) |  |  |
| Quarter 4 | 0.001 | (0.004) | 0.001 | (0.004) |  |  |
| Quarter 5 | -0.009** | (0.004) | -0.012*** | (0.004) |  |  |
| Quarter 6 | -0.006 | (0.004) | -0.005 | (0.003) |  |  |
| Quarter 7 | 0.0001 | (0.004) | -0.002 | (0.004) |  |  |
| Quarter 8 | -0.005*** | (0.004) | -0.008** | (0.004) |  |  |
| Quarter 9 | -0.012*** | (0.004) | -0.014*** | (0.004) |  |  |
| Quarter 10 | -0.005 | (0.004) | -0.012*** | (0.003) |  |  |
| Quarter 11 | -0.009** | (0.004) | -0.010*** | (0.003) |  |  |
| Quarter 12 | -0.005 | (0.004) | -0.006* | (0.003) |  |  |
| Quarter 13 | -0.006 | (0.004) | -0.010** | (0.004) |  |  |
| Quarter 14 | -0.008 | (0.004) | -0.011** | (0.004) |  |  |
| Quarter 15 | -0.006 | (0.004) | -0.009** | (0.004) |  |  |
| Quarter 16 | -0.0003 | (0.004) | -0.002 | (0.004) |  |  |
| **Outcome *k* periods prior to BPT introduction:** | | |  |  |  |  |
| k=12 |  |  |  |  | -0.015 | (0.051) |
| k=11 |  |  |  |  | 0.073* | (0.047) |
| k=10 |  |  |  |  | 0.095* | (0.048) |
| k=9 |  |  |  |  | -0.017 | (0.044) |
| k=8 |  |  |  |  | 0.021 | (0.048) |
| k=7 |  |  |  |  | 0.030 | (0.049) |
| k=6 |  |  |  |  | 0.056 | (0.048) |
| k=5 |  |  |  |  | -0.042 | (0.049) |
| k=4 |  |  |  |  | 0.093 | (0.051) |
| k=3 |  |  |  |  | 0.057 | (0.053) |
| k=2 |  |  |  |  | 0.040 | (0.060) |
| k=1 |  |  |  |  | 0.095 | (0.054) |
| Intercept | 0.086 | (0.087) | 0.146 | (0.093) | 0.216 | (0.164) |
| No. of observations | 1776 |  | 1984 |  | 444 |  |

**Table A3: Monte Carlo simulation: Varying the level of serial correlation**

| Scenario | Description | Periods: | Bias (%) | | | RMSE | | |
| --- | --- | --- | --- | --- | --- | --- | --- | --- |
|  |  |  | 3 | 10 | 30 | 3 | 10 | 30 |
|  |  |  |  |  |  |  |  |  |
|  |  |  |  |  |  |  |  |  |
| C | Parallel Trends fails + Serial correlation  ($\rho$ = 0.7) | DiD | 126 | 57 | 133 | 13 | 6 | 13 |
|  |  | Synthetic controls | 23 | 17 | 20 | 6 | 4 | 5 |
|  |  | LDV | 5 | -3 | -4 | 1 | 1 | 1 |
|  |  | Matching + DiD | 29 | 12 | 21 | 4 | 2 | 3 |
|  |  |  |  |  |  |  |  |  |
| C1 | Parallel Trends fails + Serial correlation  ($\rho$ = − 0.7) | DiD | 129 | 56 | 131 | 14 | 7 | 14 |
|  |  | Synthetic controls | 158 | 62 | 76 | 23 | 13 | 13 |
|  |  | LDV | 140 | 14 | 0.4 | 15 | 5 | 5 |
|  |  | Matching + DiD | 141 | 34 | 44 | 17 | 9 | 9 |
|  |  |  |  |  |  |  |  |  |
| C2 | Parallel Trends fails + Serial correlation  ($\rho$ = 0.4) | DiD | 126 | 57 | 133 | 13 | 6 | 13 |
|  |  | Synthetic controls | 39 | 25 | 26 | 8 | 6 | 6 |
|  |  | LDV | 20 | 1 | -3 | 3 | 2 | 2 |
|  |  | Matching + DiD | 42 | 15 | 23 | 5 | 3 | 4 |
|  |  |  |  |  |  |  |  |  |
| C3 | Parallel Trends fails + Serial correlation  ($\rho$ = − 0.4) | DiD | 128 | 56 | 131 | 13 | 6 | 13 |
|  |  | Synthetic controls | 118 | 47 | 54 | 18 | 10 | 10 |
|  |  | LDV | 98 | 10 | -0.5 | 11 | 4 | 4 |
|  |  | Matching + DiD | 105 | 25 | 33 | 13 | 7 | 7 |
|  |  |  |  |  |  |  |  |  |

**Table A4: Monte Carlo simulation: Varying the specification for the effect of unobserved confounders**

| Scenario | Description | Periods: | Bias (%) | | | RMSE | | |
| --- | --- | --- | --- | --- | --- | --- | --- | --- |
|  |  |  | 3 | 10 | 30 | 3 | 10 | 30 |
|  |  |  |  |  |  |  |  |  |
| E | Parallel Trends fails  λ_t_ has a quadratic  rather than linear trend | DiD | 150 | 132 | 325 | 15 | 13 | 33 |
|  |  | Synthetic controls | 51 | 33 | 38 | 12 | 9 | 9 |
|  |  | LDV | 23 | -2 | -7 | 4 | 3 | 3 |
|  |  | Matching + DiD | -4 | -2 | 51 | 5 | 4 | 8 |
|  |  |  |  |  |  |  |  |  |
| F | Parallel Trends fails  λ_t_ is a constant but changes in post-treatment period | DiD | 769 | 727 | 810 | 77 | 73 | 81 |
|  |  | Synthetic controls | 230 | 122 | 91 | 25 | 14 | 11 |
|  |  | LDV | 157 | 51 | 22 | 16 | 6 | 4 |
|  |  | Matching + DiD | 91 | 100 | 160 | 13 | 12 | 18 |
|  |  |  |  |  |  |  |  |  |

# Online Appendix C – Tests for identifying assumptions

We can test for the Assumption A1 (A1: Parallel trends) by estimating the following regression on the pre-treatment observations.

$$Y_{it}=X_{it}\beta+{\lambda\mu}_{i}+\delta_{t}+\theta_{t}\delta_{t}D_{it}+ \tau D_{it}+\varepsilon_{it} , \forall t\leq T_{0}$$

If the parallel trends assumption holds, the coefficients ($\theta_{t}$) on the interactions between each period-specific fixed effect and the treatment dummy ($\delta_{t}D_{it}$) should be 0. We can carry out this test using an F-test of the joint significance of $\theta_{t}$ across all pre-treatment periods. If we fail to reject the null hypothesis of parallel trends in the pre-treatment period, this increases our confidence that the parallel trends assumption holds in the post-treatment period also.

A similar indicative test is possible for Assumption A2 (A2: Independence conditional on past outcomes) can be conducted following Imbens and Wooldridge (2009, p49). We estimate an LDV model using the last pre-treatment period as the ‘treatment’ period

$$Y_{it}=X_{it}\beta+\sum_{k=1}^{T_{0}-1} \theta_{k}Y_{ik}+ \tau D_{i}+\nu_{it} , \forall t<T_{0}$$

If independence conditional on past outcomes holds in the pre-treatment period, the coefficient ($\tau$) on the treated dummy ($D_{i}$) will be 0.This can be tested using a t-test. If we fail to reject the null hypothesis of independence conditional on past outcomes in the pre-treatment period, this increases our confidence that the assumption holds in the in the post-treatment period also

The logic underlying both tests is similar: if an assumption holds in the pre-treatment period, the test assumes it continues to hold in the post-treatment periods. In both cases, a rejection of the null constitutes evidence against the particular identifying assumption.

Table A5 presents the results of these tests for our case study. The tests of assumption A1 (column 2) rejects parallel trends for mortality, while failing to reject parallel trends for the other endpoints including surgery. In contrast, the test of assumption A2 (column 3) rejects independence conditional on past outcomes for surgery but not for mortality, supporting our view that estimates based on the alternative methods are more credible than those based on DiD for this outcome.

**Table A5: Tests of identifying assumptions**

|  | **Test of**  **parallel trends**  F-statistic  (p-value) | **Test of independence conditional on past outcomes**  t-statistic  (p-value) |
| --- | --- | --- |
| Surgery within 48 hours | 0.46  (p=0.9255) | 0.038***  (p=0.001) |
| Dead within 30 days | 1.87**  (p=0.0392) | 0.001  (p=0.791) |
| Emergency  re-admissions, 30 days | 0.48  (p=0.919) | 0.002  (p=0.633) |
| Usual residence,  56 days | 1.49  (p=0.130) | 0.457  (p=0.210) |
